# Simultaneous coronary artery bypass grafting and carotid endarterectomy can be performed with low mortality rates

**DOI:** 10.5830/CVJA-2014-018

**Published:** 2014-06

**Authors:** Ebuzer Aydin, Yucel Ozen, Sabit Sarikaya, Ismail Yukseltan

**Affiliations:** Kartal Kosuyolu Training and Research Hospital, Istanbul, Turkey; Kartal Kosuyolu Training and Research Hospital, Istanbul, Turkey; Kartal Kosuyolu Training and Research Hospital, Istanbul, Turkey; Taksim German Hospital, Istanbul, Turkey

**Keywords:** coronary artery bypass grafting, carotid endarterectomy, carotid artery disease, coronary artery disease, cerebrovascular event

## Abstract

**Introduction:**

There is controversy over the best approach for patients with concomitant carotid and coronary artery disease. In this study, we report on our experience with simultaneous carotid endarterectomy (CEA) and coronary artery bypass graft (CABG) surgery in our clinic in the light of data in the literature.

**Methods:**

Between January 1996 and January 2009, a total of 110 patients (86 males, 24 females; mean age 65.11 ± 7.81 years; range 44–85 years), who were admitted to the cardiovascular surgery clinic at our hospital, were retrospectively analysed. All patients underwent simultaneous CEA and CABG. Demographic characteristics of the patients and a history of previous myocardial infarction (MI), hypertension, diabetes mellitus, hyperlipidaemia, peripheral arterial disease and smoking were recorded.

**Results:**

One patient (0.9%) with major stroke died due to ventricular fibrillation. Peri-operative neurological complications were observed in seven patients (6%). Complications were persistent in two patients. Four patients (3%) had postoperative major stroke, whereas three patients (2%) had transient hemiparesis. No peri-operative myocardial infarction was observed.

**Conclusion:**

Simultaneous CEA and CABG can be performed with low rates of mortality and morbidity.

## Abstract

There is controversy over the best approach for patients with concomitant carotid and coronary artery disease.^1^ Therapeutic strategies include isolated coronary artery bypass grafting (CABG), staged carotid endarterectomy (CEA) and CABG, reversed staged CEA and CABG, and simultaneous procedures under single anaesthesia.^2^

Although reported experiences over three decades are available, combining CEA with CABG remains to be elucidated.^3^ Furthermore, risk of cerebrovascular accident (CVA), which is one of the major predictors of prognosis of CABG, has been reported to increase up to 14% in patients with severe carotid artery stenosis (> 80%).^4^-^9^

Peri-operative neurological events such as stroke after CABG are the major neurological complications, which increase with age.^10^ The incidence of peri-operative stroke has been well documented at approximately 2% of all cardiac surgeries.^11^ Despite reduced overall complication rates over the years after CABG, the incidence of stroke remains relatively unchanged.^10^

The aetiology of peri-operative stroke is multi-factorial including hypotension or hypoperfusion-induced reduced brain flow, atherosclerosis due to micro- or macro-embolisation, and intra- or extra-cranial vascular diseases.^5^ In addition, carotid artery disease is a critical factor; however, it is considered unlikely to be the only culprit for peri-operative strokes.^12^

Although no consensus on the optimal management of patients with concomitant carotid and coronary artery disease has been reached,^13^ simultaneous CEA and CABG surgery is often associated with low rates of mortality and morbidity.^14^-^17^ In this study, we report our experience with simultaneous CEA and CABG surgery in our clinic in the light of data in the literature.

## Methods

This retrospective study included a total of 110 patients admitted to the cardiovascular surgery clinic of the Universal Taksim German Hospital between January 1996 and January 2009. All patients underwent simultaneous CEA and CABG. Demographic characteristics of the patients as well as a history of previous myocardial infarction (MI), hypertension, diabetes mellitus, hyperlipidaemia, peripheral arterial disease and smoking were recorded. Carotid artery stenosis was examined using carotid Doppler ultrasound.

Patients aged ≥ 65 years with peripheral artery disease, previous cerebrovascular disease or CEA, symptomatic disease and heart murmur were candidates for carotid Doppler ultrasound. Half of the patients underwent a shunting procedure. We performed CEA in patients with ≥ 80% carotid artery stenosis.

All patients were on acetylsalicylic acid and clopidogrel postoperatively. CEA was performed under general anaesthesia before CABG. The operation was carried out without shunting in patients with unilateral lesions and with shunting in those with bilateral critical stenosis or 100% stenosis unilaterally.

Patients with bilateral critical carotid lesions underwent surgery for unilateral carotid lesion three days prior to CEA. The remaining lesion was operated on during CABG. The arteriotomy was closed using a patch in 108 patients (98.2%), except for two patients (1.8%) who underwent primary closure. Bleeding control was achieved. The incision was left open.

Following the median sternotomy, on-pump CABG was performed with systemic hypothermia maintained at 32°C using two-stage venous and aortic cannulae. Mean arterial pressure was maintained at 60 mmHg during cardiopulmonary bypass (CPB). Operative findings are presented in Table 1.

**Table 1:** Operative findings

*Findings*	*Numerical values*
Number of distal anastomoses	296
Grafted patient rate (%)	2.69
CPB time (min)	51.72 ± 17.15 (23–95)
ACC time (min)	24.39 ± 8.41 (11–46)
Duration of carotid clamping (min)	18.56 ± 7.23 (9–42)
Carotid closure technique [*n*, (%)]	
Primary	2 (1.8)
Vein	50 (45.5)
Hemashield	58 (52.7)

CPB, cardiopulmonary bypass time; ACC, aortic cross-clamping time.

## Statistical analysis

Statistical analysis was performed using SPSS for Windows v 13.0 software (SPSS Inc, Chicago, IL, USA). Descriptive statistics were used to summarise both quantitative data including the mean, standard deviation, and maximum and minimum values, and categorical variables including frequency distribution and percentage.

Kolmogorov–Smirnov and Shapiro–Wilks tests were used to analyse normally distributed variables. One-way analysis of variance (ANOVA) was performed to determine the significance of differences between the groups. The chi-square test was also used to identify possible correlations among the variables, while the Spearman test was carried out to calculate power and direction of the correlation. A 95% confidence interval (CI) was calculated. A *p*-value of < 0.05 was considered statistically significant.

## Results

A total of 86 patients (78.2%) were male and 24 (21.8%) were female. The mean age was 65.11 ± 7.81 years (range 44–85 years). Demographic characteristics and clinical data of the patients are shown in Table 2.

**Table 2:** Demographic and clinical characteristics

*Characteristics*	*Number of patients (n)*	*Percentage (%)*
Gender		
Female	24	78.2
Male	86	21.8
Previous myocardial infarction	28	25.5
Neurological history		
Asymptomatic	82	74.5
Symptomatic	27	24.5
Hypertension	77	70
Smoking	38	34.5
Diabetes mellitus	39	35.5
Hyperlipidaemia	34	30.9
Peripheral artery disease	19	17.3

Mean age 65.11 ± 7.81 years, range 44–85

Of these patients, 27 were symptomatic, while 83 were asymptomatic. All patients who were operated on had ipsilateral carotid artery disease. Only four patients had ≥ 80% contralateral carotid artery stenosis. One patient (0.9%) with a cerebrovascular accident died due to subsequent ventricular arrhythmia in the early phase. Four patients (3%) had postoperative major stroke, whereas three (2%) had transient hemiparesis. No perioperative MI was observed. Early postoperative complications are summarised in Table 3.

**Table 3:** Postoperative complications

*Complications*	*Number*	*Percentage*
Early mortality	1	0.9
Persistent hemiplegia	3	2
Transient hemiparesis	4	3
Transient ischaemic attack	1	0.9
Peri-operative myocardial infarction	0	0
Ventricular arrhythmia	2	1
Atrial fibrillation	3	2

Spearman’s correlation showed a positive correlation between the duration of cross-clamping and shunt usage (40%). The mean duration of cross-clamping was 15.9 and 21.1 min in patients without and with shunts, respectively. It reached statistical significance (*p* < 0.049). Although shunt implantation prolonged the duration of cross-clamping, there was no statistically significant difference in neurological complication rate (*p* = 0.301).

In addition, four patients had haematoma and neurological complications due to local bleeding. One patient required re-do CABG surgery. Another patient underwent revision surgery due to sternal dehiscence. Three patients underwent revision surgery due to bleeding following CABG.

## Discussion

Today individuals with concomitant carotid and coronary artery disease are still challenging patients for surgeons. With increasing age of patients and stenosis rate, it is obvious that this will become more significant in future years.^7^ The incidence of carotid stenosis has been estimated as 12% in patients with coronary artery disease.^4^,^18^ Nearly half of the patients with carotid artery disease also have concomitant coronary artery disease.^19^

Peri-operative prevention of myocardial and cerebral accidents is an ongoing debate. Several studies have demonstrated a mortality rate for simultaneous CEA and CABG of 0–8.9% and a stroke rate of 0–9%.^4^-^9^,^19^ Lower mean arterial pressures of cardiopulmonary bypass, systemic vasodilatory response and plaque embolism during aortic cross-clamping increase the risk of peri-operative stroke in CABG patients.^20^

In a randomised study, Roach *et al.*^21^ reported a neurological complication rate of 6.1% in patients who underwent elective CABG surgery, with serious complications in 3%. The authors highlighted the critical role of examination of the carotid artery stenosis pre-operatively.

There are also several studies showing a neurological accident rate of 7.4–20.3% with a mortality rate of 6.9–13.8% in patients with concomitant carotid artery disease undergoing CABG alone.^5^,^22^,^23^ In addition, as higher morbidity rates (7–8%), which were strongly associated with peri-operative MI were reported in patients requiring CABG with isolated CEA procedure,^22^ simultaneous CEA and CABG is currently usually recommended.^22^,^25^

Furthermore, there is controversy on the optimal treatment for patients with concomitant carotid and coronary artery disease. Surgeons should consider a number of clinical parameters when selecting the simultaneous or staged approach. To illustrate, postoperative MI was reported to be 7% in symptomatic patients and 1% in asymptomatic patients with coronary artery disease who underwent CEA followed by CABG.^19^,^26^

The incidence of peri-operative stroke, on the other hand, is markedly increased in patients with ≥ 80% carotid artery stenosis, suggesting a staged approach, including CABG followed by CEA.^27^ However, the incidence of cardiovascular accidents is mainly associated with embolism rather than low cardiac-output thrombosis rate in patients undergoing elective CABG surgery.^28^

Simultaneous intervention was first described in a single-anesthesia period.^29^ Trachiotis *et al.*^30^ and Akins *et al.*^18^ reported that the simultaneous approach was highly effective in reducing neurological and myocardial complications. Additionally, Takach *et al.*^31^ indicated that simultaneous intervention was as safe as the staged approach in high-risk patients, which was consistent with our study findings.

In another single-centre study, the simultaneous approach to CEA and CABG was reported to be associated with equivalent mortality and stroke profiles, as well as lower overall complication rates and hospital charges.^20^ There are several studies reporting a shorter length of hospital stay, 26,32 lower costs^26^,^33^ and acceptable early mortality and morbidity rates.^34^-^36^ Similarly, we found the mortality and major stroke rates to be 0.9 and 2%, respectively.

Nevertheless, despite an increased number of studies showing the merits of the simultaneous approach, national and international guidelines have provided no consensus yet due to the lack of prospective, randomised clinical trials. However, simultaneous CEA and CABG in asymptomatic patients with bilateral severe disease in particular, has been widely recommended.^13^

Moreover, carotid artery stenting (CAS), which is less invasive, with a lower rate of myocardial events, has been popular in recent years. However, deliberate action should be taken until its long-term results are documented, as there is currently limited evidence supporting the use of CAS.^10^

## Conclusion

Our clinical experience indicated that simultaneous CEA and CABG can be performed safely. Furthermore, it increases patient comfort, since anaesthesia is given once, and two operations are carried out at a single session. Therefore, we recommend the simultaneous approach for patients with coronary and carotid artery disease. However, further large-scale, multicentred, randomised clinical studies are required to draw final conclusions.
